# Supplementary material for: Colorectal Cancer Presentation and Survival in Young Individuals: A Retrospective Cohort Study
Source: Cancers (Basel). 2018 Nov 28;10(12):472. doi: 10.3390/cancers10120472 (PMC6316605; doi:10.3390/cancers10120472)
Supplement: Supplementary file 1 [file cancers-10-00472-s001.pdf]

Supplementary Materials: Colorectal Cancer Presentation and Survival in Young Individuals: A Retrospective Cohort Study

Mark B. Ulanja, Bryce D. Beutler, Mohit Rishi, Chioma Ogala, Darryll R. Patterson, Nageshwara Gullapalli and Santhosh Ambika

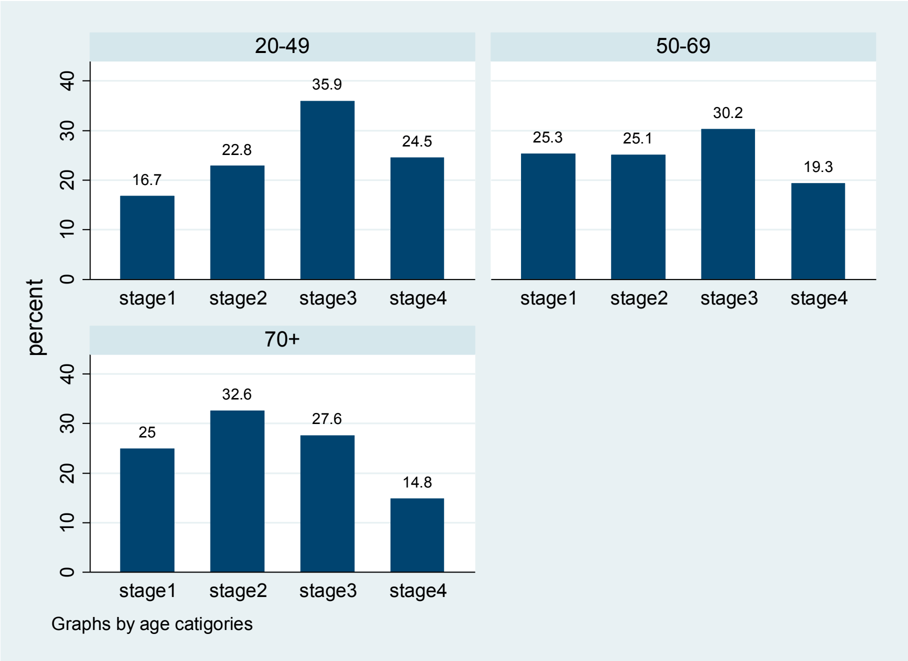

Figure S1. CRC stages and distribution by age group. 20–49 = age group 20–49, 50–69 = age group 50 to 69, 70+ = 70 years and older; stage = CRC stages.

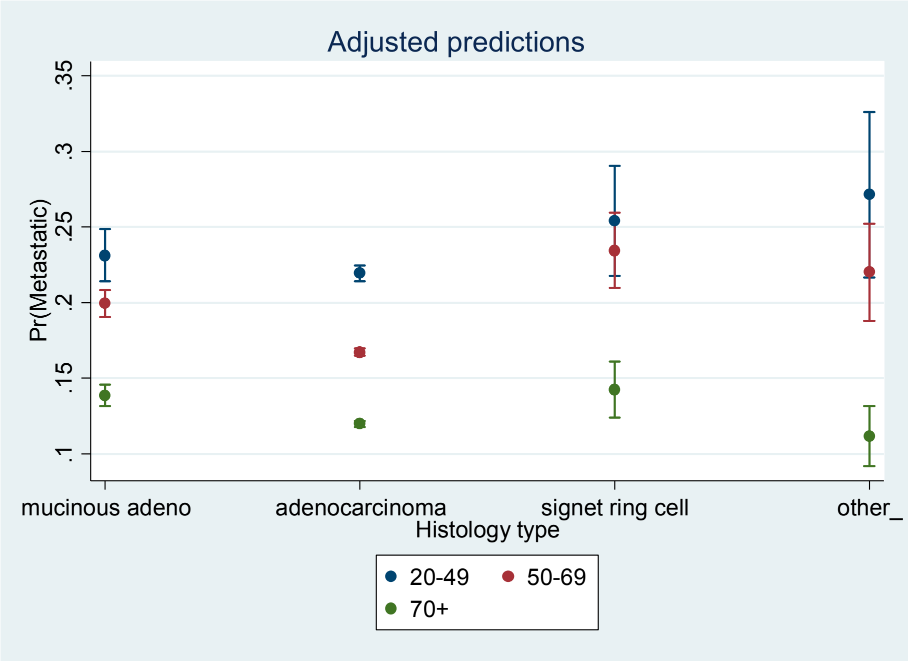

Figure S2. Predicted probabilities for age groups by CRC histology type. 20–49 = age group 20 to 49, 50–69 = age group 50 to 69, 70+ = 70 years and older; Pr (metastatic) = probability of presenting with

stage IV disease; Mucinous adeno = mucinous adenocarcinoma; adenocarcinoma = adenocarcinoma non otherwise specified; signet ring cell = signet ring cell carcinoma; and other = medullary carcinoma; enosquamous adenocarcinoma and mixed adenocarcinoma.

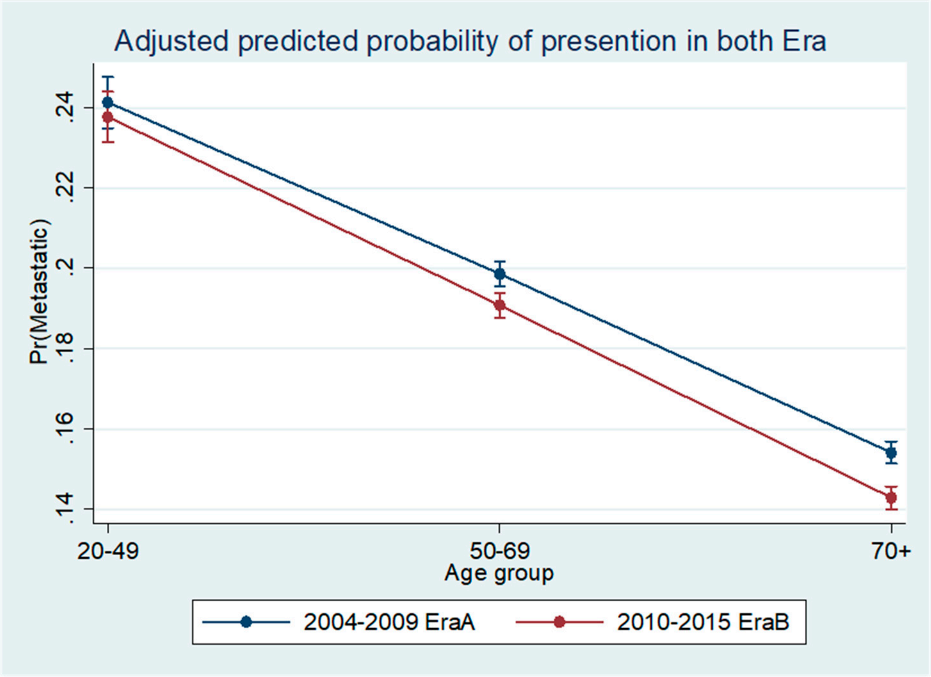

Figure S3. Adjusted Predicted probabilities of metastatic disease by age for EraA and EraB. Era A is 2004 to 2009; Era B is 2010 to 2015; Pr (metastatic) = probability of presenting with stage 4 disease.

Table S1. Hazard ratios (HR) of all-cause and CRC specific mortality in different age groups stratify by histology. HR= hazard ratio; NOS = Non Otherwise Specified.

Colorectal Cancer	Age Groups		
	20–49	50–69	70 and older
**All-cause mortality**			
**Mucinous adenocarcinoma**			
Adjusted HR	Reference (20–49)	1.12 (1.04–1.21)	1.73 (1.61–1.87)
**Adenocarcinoma NOS**			
Adjusted HR	Reference (20–49)	1.17 (1.14–1.20)	2.06 (2.01–2.11)
**Signet ring cell carcinoma**			
Adjusted HR	Reference (20–49)	**1.20 (1.05–1.38)**	**1.43 (1.23–1.66)**
**Other**			
Adjusted HR	Reference (20-49)	1.18 (0.93–1.50)	1.81 (1.42–2.31)
**CRC-Specific Mortality**			
**Mucinous adenocarcinoma**			
Adjusted HR	Reference (20–49)	1.01 (0.93–1.10)	1.20 (1.11–1.31)
**Adenocarcinoma NOS**			
Adjusted HR	Reference (20–49)	1.05 (1.02–1.08)	1.46 (1.42–1.50)
**Signet ring cell carcinoma**			
Adjusted HR	Reference (20–49)	1.13 (0.99–1.31)	1.16 (0.99–1.36)
**Other**			
Adjusted HR	Reference (20–49)	1.12 (0.88–1.44)	1.47 (1.13–1.91)

© 2018 by the authors. Licensee MDPI, Basel, Switzerland. This article is an open access article distributed under the terms and conditions of the Creative Commons Attribution (CC BY) license (http://creativecommons.org/licenses/by/4.0/).
